# Red Ginseng Improves Exercise Endurance by Promoting Mitochondrial Biogenesis and Myoblast Differentiation

**DOI:** 10.3390/molecules25040865

**Published:** 2020-02-16

**Authors:** Eun Ju Shin, Seongin Jo, Sungbin Choi, Chang-Won Cho, Won-Chul Lim, Hee-Do Hong, Tae-Gyu Lim, Young Jin Jang, Mi Jang, Sanguine Byun, Youngkyung Rhee

**Affiliations:** 1 Korea Food Research Institute, Wanju-gun, Jeollabuk-do 55365, Korea; sej296@naver.com (E.J.S.); cwcho@kfri.re.kr (C.-W.C.); 07934@kfri.re.kr (W.-C.L.); honghd@kfri.re.kr (H.-D.H.); tglim83@kfri.re.kr (T.-G.L.); jyj616@kfri.re.kr (Y.J.J.); jangmi@kfri.re.kr (M.J.); 2 Division of Bioengineering, Incheon National University, Incheon 22012, Korea; friend9698@naver.com (S.J.); 201921148@inu.ac.kr (S.C.); 3 Department of Food Science & Biotechnology, Sejong University, Seoul 05006, Korea

**Keywords:** red ginseng, exercise endurance, mitochondrial biogenesis, myoblast differentiation

## Abstract

Red ginseng has been reported to elicit various therapeutic effects relevant to cancer, diabetes, neurodegenerative diseases, and inflammatory diseases. However, the effect of red ginseng on exercise endurance and skeletal muscle function remains unclear. Herein, we sought to investigate whether red ginseng could affect exercise endurance and examined its molecular mechanism. Mice were fed with red ginseng extract (RG) and undertook swimming exercises to determine the time to exhaustion. Animals fed with RG had significantly longer swimming endurance. RG treatment was also observed to enhance ATP production levels in myoblasts. RG increased mRNA expressions of mitochondrial biogenesis regulators, *NRF-1*, *TFAM*, and *PGC-1α*, which was accompanied by an elevation in mitochondrial DNA, suggesting an enhancement in mitochondrial energy-generating capacity. Importantly, RG treatment induced phosphorylation of p38 and AMPK and upregulated PGC1α expression in both myoblasts and in vivo muscle tissue. In addition, RG treatment also stimulated C2C12 myogenic differentiation. Our findings show that red ginseng improves exercise endurance, suggesting that it may have applications in supporting skeletal muscle function and exercise performance.

## 1. Introduction

The maintenance of skeletal muscle function is an important factor in controlling the quality of life. Conversely, reductions in muscle function can contribute to adverse health outcomes including disability, frailty, fatigue, insulin resistance, and mortality [1,2,3,4,5]. Therefore, the prevention of muscle loss and enhancing muscle function is of significant relevance not only for athletes but also for individuals seeking to maintain a healthy body. Muscle loss can occur for a variety of causes, including acute or chronic diseases, age-related molecular changes, malnutrition, or inactivity [6]. Although physical exercise and balanced nutrition is the gold standard for maintaining skeletal muscle health, recent research shows that consumption of nutraceuticals may also help to improve muscle function and strength [7]. Hence, the development of novel agents that can promote muscle mass/function could provide additional strategies to support muscle health. 

Skeletal muscle function is tightly controlled by a complex interplay of signaling pathways. Peroxisome proliferator-activated receptor gamma co-activator 1α (PGC-1α) is a master transcription coactivator that regulates mitochondrial energy metabolism and muscle function, and its expression is reduced with aging and increased by exercise training [8,9,10]. PGC-1α is responsible for the changes seen after endurance training, such as mitochondrial biogenesis, oxidative muscle fiber formation, and angiogenesis, which contribute to an overall increase in exercise capacity [11,12]. PGC-1α overexpression in muscle cells increases mitochondrial DNA copy number and respiratory capacity [9], while skeletal muscle-specific PGC-1α knockout has been reported to reduce oxidative muscle fibers and mitochondrial respiration [9]. 

AMP-activated protein kinase (AMPK) is another central regulator of skeletal muscle metabolism and function [13]. AMPK is similarly phosphorylated and activated by exercise training in both humans and rodents [9]. AMPK is required for mitochondrial biogenesis in skeletal muscle and regulates exercise capacity [14,15]. Deficiencies in AMPK result in reduced running capacity in mice, while the treatment of the AMPK activator AICAR promotes exercise endurance and mitochondrial activity by increasing muscle fiber regeneration [16].

Ginseng (*Panax ginseng* Meyer, Araliaceae) is a medicinal herb that has been shown to exhibit various bioactive properties [17,18]. Red ginseng (RG) is generated by repeating multiple cycles of steaming and drying raw ginseng. Evidence suggests that there are numerous superior bioactivities present in RG compared to white ginseng [19,20]. The various health-promoting effects of RG have been reported to include alleviation of fatigue, protection from muscle damage after strenuous exercise, and improvements in energy metabolism [21,22,23,24]. Red ginseng has been reported to show an increase in multiple types of ginsenosides compared to white ginseng, including Rg3, Rk1, Rf, and Rg5 [25,26]. Rg3 was previously reported to display anti-fatigue effects [27]. Rg1, Rb1, and Rb2 were reported to promote myobloast differentiation [28,29]. However, the precise mechanisms of RG on exercise endurance and muscle function are not fully understood. Also, since many people consume red ginseng extract rather than individual ginsenosides, the effect of mixtures of ginsenosides in the form of ginseng extract can be of key interest for the public. In the current study, we sought to investigate the effect of red ginseng on muscle function and tested its impact on exercise capacity in vivo.

## 2. Results

### 2.1. Administration of RG Improves Swimming Performance in Mice

**Figure 1:**
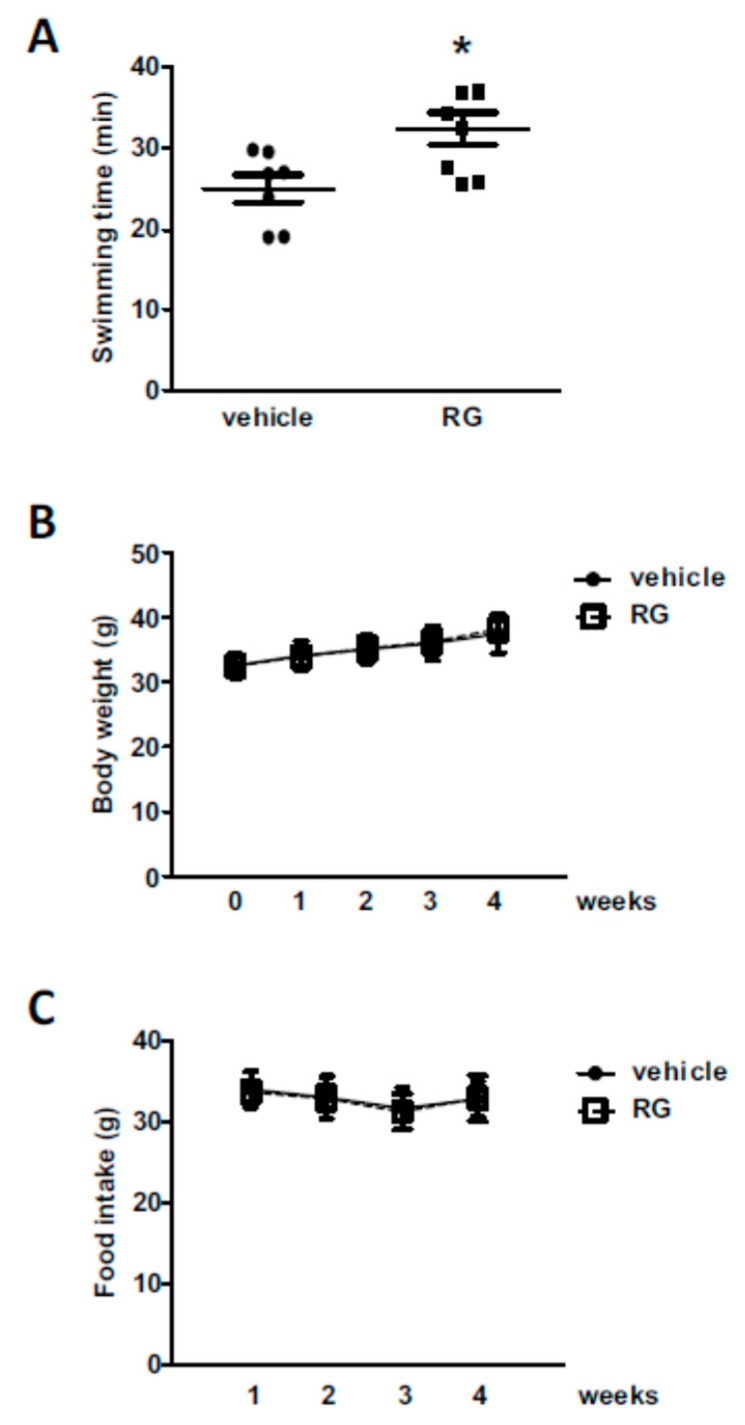
Effects of red ginseng (RG) on exhaustive swimming time. Mice were fed with vehicle or 100 mg/kg RG for 28 days, and (**A**) exhaustive swimming test was performed. Exhaustive swimming time was recorded as the time when each mouse was unable to return to the surface to breathe within 7 s. (**B**) Bodyweight and (**C**) food intake were measured. Data were expressed as mean ± S.E. (n = 8). * *p* < 0.05 versus vehicle group.

We employed the exhaustive swimming test model to evaluate exercise performance in mice [30]. The concentration of RG for the animal experiment was chosen based on previous reports [31,32,33]. The animals were fed with 100 mg/kg of RG for 28 days and assessed for swimming endurance. RG supplementation increased swimming time by approximately 30% compared to the vehicle-treated control group (24.92 ± 1.61 min and 32.36 ± 1.97 min, respectively, Figure 1a). There were no noticeable changes in body weight or food intake level associated with RG supplementation (Figure 1B,C).

### 2.2. RG Increases ATP Production Levels in C2C12 Myoblasts

**Figure 2:**
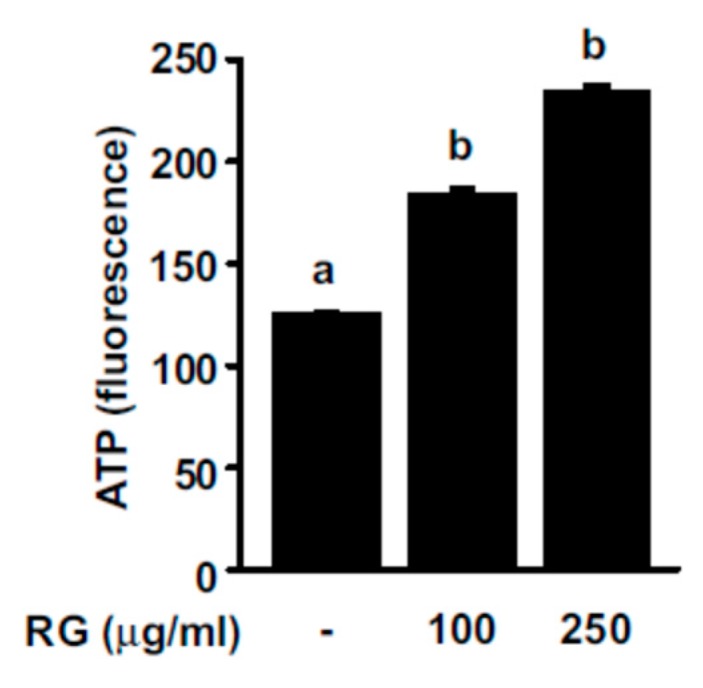
Effects of RG on ATP levels in C2C12 myoblasts. C2C12 cells were induced to differentiate for 2 days, and then cells were treated with 100 or 250 μg/mL of RG for 24 h. ATP levels were measured by ATP determination kit. Bars with different letters mean significant differences from each other (*p* < 0.05).

More frequent muscle contractions require a higher ATP supply compared to the resting state, and an increase in ATP production can help improve exercise performance. To examine the effect of RG on ATP production, we measured ATP levels after treating C2C12 myoblasts with RG. RG treatment elicited a dose-dependent increase in ATP levels in myoblasts (Figure 2). 

### 2.3. RG Promotes Mitochondrial Biogenesis in C2C12 Myoblasts

**Figure 3:**
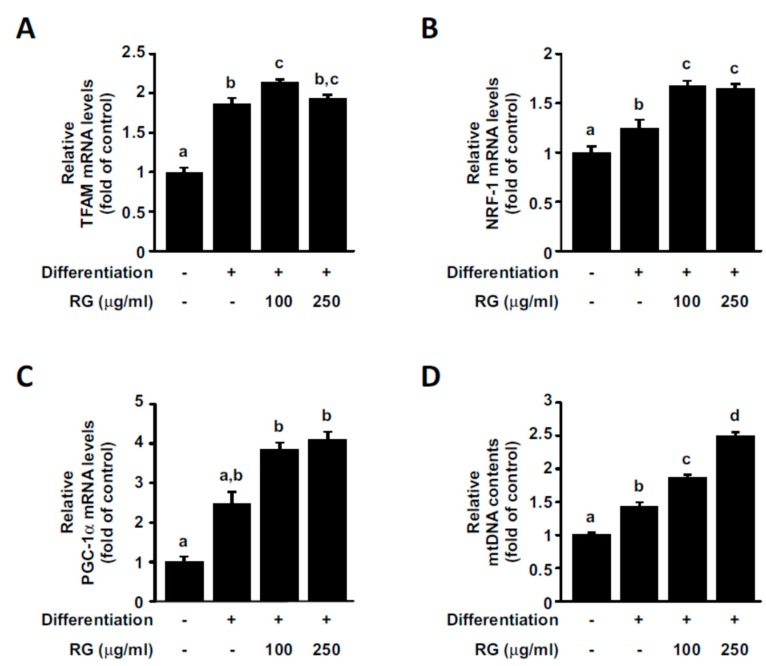
Effects of RG on mitochondrial biogenesis. Cells were induced to differentiate for 2 days, and then cells were treated with 100 or 250 μg/mL of RG for 48 h. (**A**–**C**) Relative mRNA expressions of nuclear respiratory factor 1 (NRF-1), mitochondrial transcription factor A (TFAM), and PPAR gamma coactivator 1-alpha (PGC-1α) were detected using real-time PCR analysis. The expression levels were normalized to that of GAPDH. (**D**) Mitochondrial DNA (mtDNA) copy number was measured. Data are expressed as mean ± S.D. Bars with different letters mean significant differences from each other (*p* < 0.05).

Mitochondrial biogenesis can contribute to increased oxidative phosphorylation capacity, which, in turn, can lead to a rise in ATP production levels. Mitochondrial transcription factor A (TFAM) promotes the transcription and replication of mitochondrial DNA, playing an essential role in mitochondrial biogenesis [34]. Nuclear respiratory factor-1 (NRF-1) is a transcription factor that contributes to the upregulation of several key mitochondrial proteins, including TFAM [34]. We observed that the treatment of C2C12 myoblasts with RG increased *TFAM* and *NRF-1* gene expression levels (Figure 3A,B). PGC-1α functions as a central regulator of mitochondria function and promotes mitochondrial biogenesis by regulating various transcription factors, including NRF-1 [9]. *PGC-1α* mRNA levels were upregulated in a dose-dependent manner by RG treatment (Figure 3C). To further confirm the effect of RG on mitochondrial biogenesis, we assessed mtDNA copy contents. Mitochondrial DNA content was significantly amplified after RG treatment (Figure 3D).

### 2.4. RG Upregulates p38, AMPK, and PGC-1α

**Figure 4:**
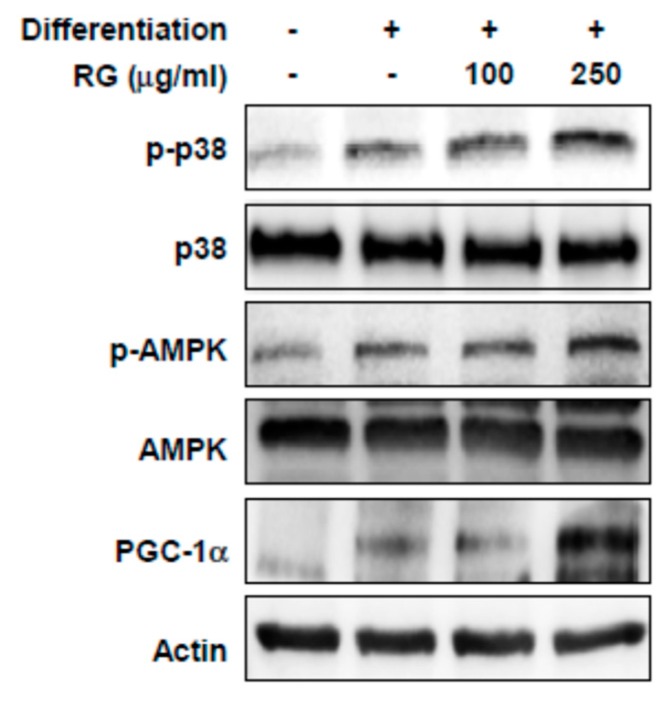
Effects of RG on phosphorylation levels of p38, AMP-activated protein kinase (AMPK) and PGC-1α in C2C12 myotubes. Cells were induced to differentiate for 2 days, and then cells were treated with 100 and 250 μg/mL of RG for 48 h. Cell lysates were immunoblotted with antibodies against phosphorylated-p38 MAPK (p-p38), phosphorylated AMPK (p-AMPK), total p38, AMPK, PGC-1α and actin as a loading control.

To investigate the molecular mechanism responsible for the RG-dependent improvement in muscle function and mitochondrial biogenesis, we examined the effect of RG on p38, AMPK, and PGC1α signaling. The presence of RG elevated the phosphorylation levels of p38 and AMPK in differentiated C2C12 myoblasts (Figure 4). In addition, PGC-1α protein expression significantly increased after RG treatment (Figure 4).

**Figure 5:**
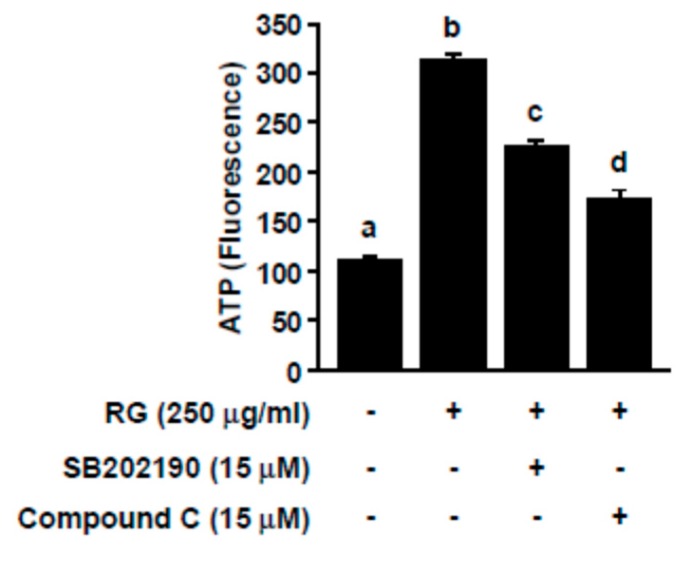
Effects of p38 and AMPK inhibition on RG-induced ATP levels in C2C12 myotubes. After differentiation, cells were treated with SB202190 (p38 inhibitor) or Compound C (AMPK inhibitor) for 30 min, prior to treatment with RG. Then, ATP levels were evaluated. Data are expressed as mean ± S.D. Bars with different letters mean significant differences from each other (*p* < 0.05).

To further assess the involvement of p38 and AMPK in RG-mediated ATP production, we co-treated SB202190 or dorsomorphin (i.e., compound C) with RG. SB202190 and dorsomorphin function as p38 and AMPK inhibitors, respectively. RG-mediated increases in ATP production were negated by SB202190 and dorsomorphin in C2C12 myoblasts (Figure 5). These data demonstrated that RG enhances ATP production through the AMPK and p38 signaling pathway.

### 2.5. RG Promotes Myogenic Differentiation

**Figure 6:**
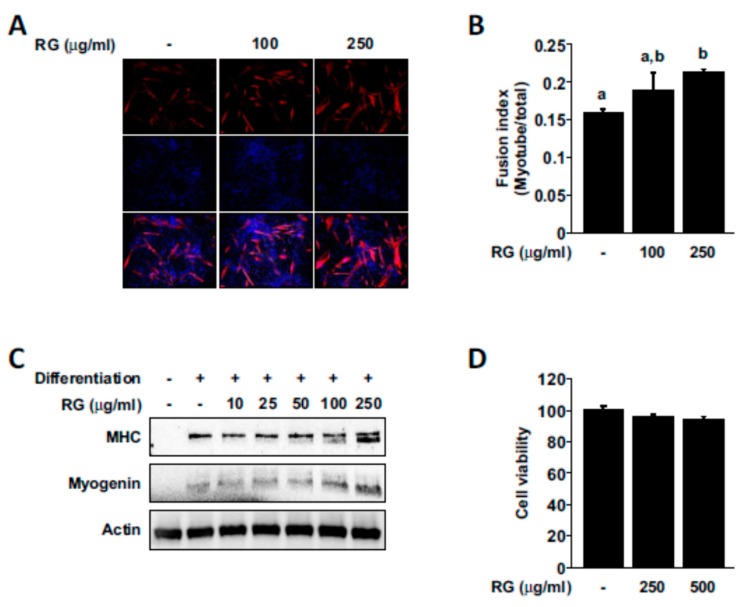
Effects of RG on myogenic differentiation. Cells were induced to differentiate for 2 days, and then cells were treated with the indicated dose of RG for 48 h. (**A**) To evaluate myotube formation, cells were stained for myosin heavy chain (MHC) (red). Nuclei were stained with DAPI (blue). (**B**) Fusion index was determined (number of nuclei within MHC-stained myotubes/total nuclei) (**C**) Protein extracts were analyzed for the levels of MHC, myogenin, and beta-actin using immunoblot analysis. (**D**) Cell viability was measured by MTS assay. Bars with different letters mean significant differences from each other (*p* < 0.05).

Increases in muscle cell growth and differentiation is known as key contributing factors that can improve physical performance [35,36], and we next examined whether RG treatment had any effect on myogenesis. RG treatment promoted myogenic differentiation and increased the fusion index (the number of nuclei inside MHC-positive myotubes divided by total number of nuclei; Figure 6A,B). We also observed that RG could upregulate the expression of MHC and myogenin (Figure 6C). RG did not cause any noticeable effect on the cell viability of differentiated C2C12 myoblasts (Figure 6D).

### 2.6. RG Administration Activates p38, AMPK, and PGC-1α Signaling In Vivo

**Figure 7:**
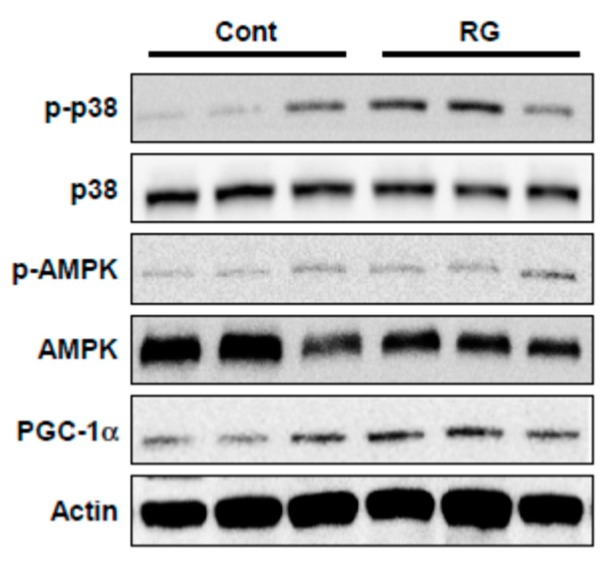
Effects of RG on protein expression in the muscles of mice fed a vehicle or RG. Mice were fed with vehicle or 100 mg/kg RG for 28 days. Muscle tissue (tibialis anterior) lysates were immunoblotted with antibodies against phosphorylated-p38 MAPK (p-p38), phosphorylated AMPK (p-AMPK), total p38, AMPK, PGC-1α, and actin as a loading control.

Since RG upregulated p38 and AMPK phosphorylation and PGC-1α expression in C2C12 myoblasts, we examined whether these signaling events could be recapitulated in vivo. RG supplementation increased the phosphorylation of p38 and AMPK as well as PGC-1α expression in mouse muscle tissue (tibialis anterior) (Figure 7).

### 2.7. Analysis of Ginsenoside Profiles for RG

**Figure 8:**
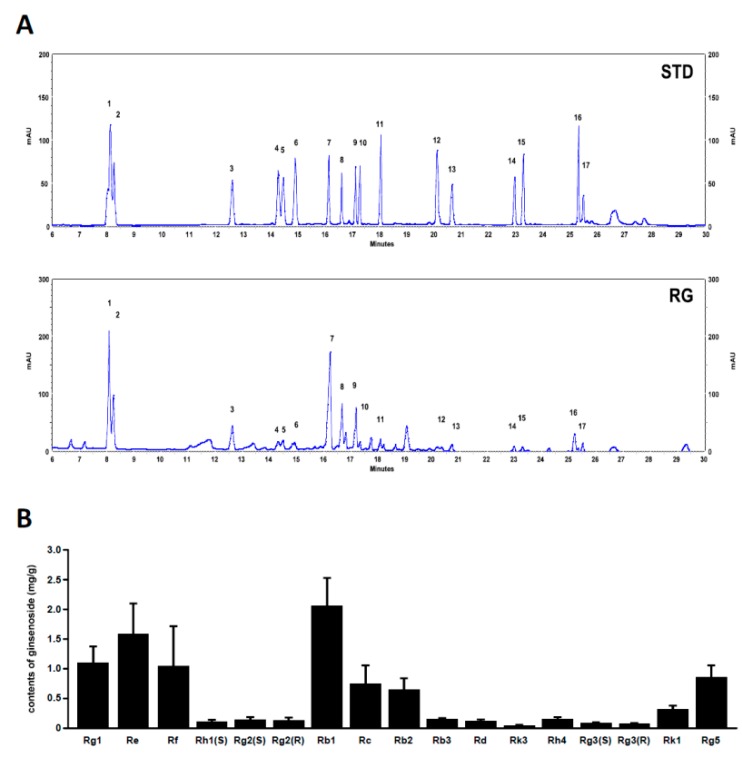
Ultra-performance liquid chromatography (UPLC) analysis of ginsenosides in RG. (**A**) The chromatograms of the standard mixture and RG. Peaks: 1; Rg1, 2; Re, 3 Rf, 4; Rh1, 5; Rg2(S), 6; Rg2(R), 7; Rb1, 8; Rc, 9; Rb2, 10; Rb3, 11; Rd, 12; Rk3, 13; Rh4, 14; Rg3(S), 15; Rg3(R), 16; Rk1, 17; Rg5. (**B**) Contents of 14 ginsenosides in red ginseng (mg/g). Characterization and quantification ginsenosides in RG were conducted using ginsenoside standards.

We analyzed the profiles of various ginsenosides (17 species) in RG using UPLC. The UPLC chromatograms of RG are shown in Figure 8A. The ginsenoside composition of RG included Rg1 1.09, Re 1.58, Rf 1.04, Rh1(S) 0.1, Rg2(S) 0.14, Rg2 (R) 0.13, Rb1 2.06, Rc 0.74, Rb2 0.64, Rb3 0.15, Rd 0.12, Rk3 0.04, Rh4 0.15, Rg3(S) 0.08, Rg3 (R) 0.07, Rk1 0.31, and Rg5 0.85 mg/g, respectively (Figure 8B). The chemical structures of ginsenosides are shown in Supplementary Figure S1. 

## 3. Discussion

While there have been studies demonstrating the effect of RG on the protection and recovery of muscle tissue after exercise, as well as the alleviation of fatigue, the direct impact on exercise performance and myoblast differentiation remains poorly understood. In the present study, we observed that RG supplementation increased the duration of swimming time for the animals tested. The improvement in exercise performance appears to be attributed to the stimulation of mitochondrial biogenesis in myoblasts, leading to a higher ATP-generating capacity. 

Several signaling pathways are known to activate PGC-1α. p38 mitogen activated protein kinase (p38 MAPK) stabilizes and activates PGC-1α protein [10,37], while p38 MAPK also increases PGC-1α transcription by phosphorylating activating transcription factor 2 (ATF2), a transcription factor of PGC-1α [38]. AMPK activation by AICAR increases *PGC-1α* mRNA expression and AMPK also activates PGC-1α through direct phosphorylation of PGC-1α [39,40]. In the present study, we found that RG increased PGC-1α mRNA and protein expression, as well as the phosphorylation of AMPK and p38 MAPK, suggesting that RG regulates PGC-1α by phosphorylating p38 MAPK and AMPK. 

PGC-1α is a master regulator of mitochondrial biogenesis, which occurs via nuclear respiratory factor (NRF1) and mitochondrial transcription factor A (TFAM), downstream transcription factors of PGC-1α. PGC-1α regulates mitochondrial DNA transcription by activating NRF1 and NRF2, which promotes the expression of TFAM [34,41]. NRF-1 and NRF-2 promote the transcription of key mitochondrial enzymes while TFAM increases the transcription and replication of mitochondrial DNA [26]. In previous studies, increased PGC-1α and mitochondrial biosynthesis have been reported to induce muscle differentiation [42,43]. Our results suggest that RG-stimulated mitochondrial biogenesis may lead to an induction of ATP production. 

Additionally, an increase in myogenesis can lead to improved exercise performance [36]. RG treatment was shown to promote myogenic differentiation in our study. To our knowledge, this is the first study to investigate the effects of RG on myoblast differentiation in C2C12 cells. 

Ginseng is mainly composed of ginsenosides, polysaccharides, phenols, and polyacetylene [44]. Among them, ginsenosides are considered to be the major ingredients responsible for the bioactivity of ginseng. Red ginseng is produced by conducting several rounds of steaming and drying white ginseng. Red ginseng contains elevated levels of a variety of characteristic ginsenosides, including (20*S*- and 20*R*-) Rg3, Rh1, Rk1, and Rg5, compared to white ginseng [45,46]. According to previous studies, 20(*S*)-ginsenoside Rg3, Rg1, and Ro improved fatigue-associated factors in vivo [27,47]. In addition, Rg1 and Rb2 were reported to induce myoblast differentiation through Akt and p38 signaling [28,29]. Rg1, Rb1, and Rg3 have been linked with an improvement in mitochondrial function [48,49]. While it is not easy to find a clear structure–activity relationship of ginsenosides against muscle function, interestingly Rb1 and Rg3, which are both from the protopanaxadiol group of ginsenosides (Supplementary Figure S1), displayed beneficial effects toward muscle function [50,51]. These observations suggest that specific ginsenosides of red ginseng can contribute to the improvement in exercise endurance and muscle function. 

We found that the administration of RG improves exercise performance by upregulating the PGC-1α-NRF1-TFAM pathway, which contributes to the production of ATP and stimulates myoblast differentiation. Collectively, our findings suggest that red ginseng extract may have utility as a functional food ingredient that promotes exercise endurance.

## 4. Materials and Methods

### 4.1. Reagents

MTS reagent, SB202190, and compound C were purchased from Sigma-Aldrich (St Louis, MO, USA). Antibodies against myosin heavy chain (MHC), Akt, p38 MAPK, and myogenin were obtained from Santa Cruz Biotechnology (Santa Cruz, CA, USA). Antibodies against phospho-Akt, phospho-p38 MAPK, and phosphor-AMPK were obtained from Cell Signaling (Beverly, MA, USA). PGC-1α antibody was purchased from Abcam (Cambridge, MA, USA).

### 4.2. Preparation of Red Ginseng Extracts

Red ginseng powder (Korean ginseng, *Panax ginseng* Meyer, Araliaceae, Cat. 1997046707571) was provided by Kuan Industrial Co., Ltd. (Republic of Korea). For water extraction, red ginseng powder (10 g) was subjected to extraction twice at 80 °C for 2 h with the 50 times the volume of distilled water using reflux condenser. After extraction, the slurry was filtered with filter paper (Whatman, Maidstone, England) and vacuum-concentrated using a rotary evaporator. Subsequently, the solid residue was dissolved in distilled water and freeze-dried for use in the experiments. The extraction yield of the RG was approximately 54.8% ± 0.9%. 

### 4.3. Animals and Treatment

ICR mice (5 weeks old, n = 16) were purchased from Doo-Yeol Biotech (Seoul, Republic of Korea), and maintained under standard environmental conditions (constant temperature with 12 h light and 12 h dark cycle) with free access to food (1314 FORTI diet (22.5% Crude protein, Altromin Spezialfutter GmbH, Germany) and water. After 1 week of adaptation, the animals were randomly assigned to two groups (eight mice/groups) for oral gavage treatment with water (control) or 100 mg/kg RG once daily for 28 days. Food intake and body weight were measured weekly. All experiments were performed according to procedures approved by the Institutional Animal Care and Use Committee (WJIACUC20190202-1-08).

### 4.4. Exhaustive Swimming Exercise Performance Test

After three weeks of feeding, the animals were encouraged to swim for 30 to 60 min to adapt to the swimming environment. One week later, the mice were placed in a swimming pool (height: 290 mm, diameter: 250 mm) with water maintained at 25 ± 2 °C. The animals were made to swim with a load attached to the tail base equal to 5% of their body weight. Exhaustive swimming time was recorded as the time when each mouse was unable to return to the surface to breathe within 7 s. The time taken until this point was defined as the exercise endurance value.

### 4.5. Cell Culture

Murine C2C12 myoblasts were purchased from the American Type Culture Collection (Mannassas, VA, USA) and maintained at 37 °C in a humidified atmosphere under 5% CO_2_. Cells were cultured in high-glucose Dulbecco’s modified Eagle’s medium (DMEM, Life Technologies, Carlsbad, CA, USA) with 10% fetal bovine serum (FBS, GIBCO, Grand Island, NY, USA) and antibiotics (Life Technologies, Carlsbad, CA, USA). Muscle differentiation was induced with differentiation medium containing 2% horse serum (GIBCO, Grand Island, NY, USA). After 2 days, the differentiated C2C12 cells (D2) were further treated with RG (100, 250 µg/mL) for an additional 2 days, before the samples were collected.

### 4.6. Measurement of Adenosine Triphosphate Levels

ATP levels were determined using an ATP fluorometric assay kit (Abcam, Cambridge, MA, USA) according to the manufacturer’s instructions. In brief, the cells were washed after treatment with cold phosphate buffered saline (PBS) and resuspended in 100 µL of ATP assay buffer. The resuspension solution was centrifuged for 5 min at 13,000 *g* (4 ℃), and 50 µL of the supernatant was collected and loaded into each well of a 96-well black plate, manufactured by SPL Life Sciences (Pocheon, Republic of Korea). A total of 50 µL of an ATP reaction mix was prepared for each reaction and added to each of the sample wells. The 96-well plate was then shaken and incubated at room temperature for 30 min while protected from light. Fluorescence was measured on a microplate reader at Ex/Em = 535/587 nm.

### 4.7. Quantitative Real-Time PCR and Analyses of mtDNA Content

Total RNA and DNA was extracted from isolated C2C12 cells using easy blue reagent and a DNeasy Kit (QIAGEN Sciences, Germantown, MD, USA), respectively. Quantitative real-time PCR was performed with SYBR green Master Mix (Toyobo, Osaka, Japan). The primer sequences were as follows: nuclear respiratory factor 1 (NRF-1) (F: 5′-CCATCTATCCGAAAGAGACAGC-3’, R:5’-GGGTGAGATGCAGAGTACAATC-3′), mitochondrial transcription factor A (tFAM) (F: 5’-GGAATGTGGAGCGTGCTAAAA-3′, R: 5’-GCTGGAAAAACACTTCGGAATA-3’), PGC-1α (F: 5’-TATGGAGTGACATAGAGT GTGCT-3’, R: 5’-CCACTTCAATCCACCCAGAAAG-3’), and GAPDH (F: 5’-CATGGCCTTCCGTGTTCCTAC-3, R: 5-TCAGTGGGCCCTCAGATGC-’3). Real time PCR was used to detect mtDNA copy number. The sequences are as follows: mitochondrial DNA (mtDNA) (F: 5′-CGTTAGGTCAAGGTGTAGCC -3′, R: 5′-CCAGA CACACTTTCCAGTATG-3′), and Actin (F: 5′-GATTACTGCTCTGGCTCCTAGC -3′, R: 5’-GATTACTGCTCTGGCTCCTAGC-3′). Gene expression was evaluated using the ΔΔCT method, with GAPDH or actin as a house keeping gene.

### 4.8. Immunoblotting

Cells were lysed in a cell lysis buffer (Cell Signaling) containing a protease inhibitor and PMSF. Protein concentrations were determined using BCA assay reagent (Thermo). Equal amounts of protein were subjected to SDS-polyacrylamide gel electrophoresis (PAGE) and transferred onto nitrocellulose membranes (Bio-Rad, Hercules, CA, USA). After blocking with 5% skim milk for 1 h, the membranes were probed with the following specific antibodies: anti-MHC, anti-myogenin, and anti-β actin. Membranes were then incubated with HRP-conjugated secondary antibodies. Protein bands were detected with a chemiluminescence detection kit (GE Healthcare, Pittsburgh, PA, USA).

### 4.9. Immunofluorescence

Cells were grown on coverslips and fixed with 4% paraformaldehyde for 15 min at room temperature. The cells were then permeabilized with 0.05% Triton X-100 for 10 min and incubated with an MHC antibody overnight at 4 °C, followed by Alexa 48-conjugated anti-mouse IgG (Thermo Fisher Scientific). The cells were then counterstained with DAPI (4′,6-diamidino-2-phenylindole). The expression of MHC was observed using fluorescence microscope (Olympus, Japan). Fusion index was measured using NIH image J software (Bethesda, MD, USA).

### 4.10. Cell Viability

Cell viability was measured under the same conditions used for myoblast differentiation in the MTS assay. Cells were incubated with MTS solution for 30 min, and optical density was determined at 450 nm (Infinite M200, Tecan Trading AG, Mannedorf, Switzerland).

### 4.11. UPLC Analysis

For UPLC analysis, ginsenoside standards and samples were dissolved in 80% methanol and sonicated for 30 min in RT. Then, supernatant solutions were filtered using a 0.2 μm syringe filter (PVDF, Whatman International Ltd., Maidstone, England) and injected directly into the UPLC system (Hitachi). The chromatographic separation was accomplished on a ACQUITY BEH C18 column (100 × 2.1 mm, 1.7 μm; waters). The binary gradient elution system consists of water (A) and acetonitrile (B). The elution gradient was as follows: 0–0.5 min (15% B), 14.5 min (30% B), 15.5 min (32% B), 18.5 min (38% B), 22.0 min (41% B), 25 min (55% B), 29 min (55% B), 33 min (70% B), 35 min (90% B), 37 min (90% B), 38 min (15 %), 41 min (15% B). The flow rate was set at 0.6 mL/min and the sample injection volume was 6 μL. For the determination of various ginsenoside contents from RG, the standard stock solution was analyzed together with the samples, and analyzed concentrations in the samples were estimated by peak areas. All samples were analyzed in triplicate.

### 4.12. Statistical Analysis

Data are expressed as the means ± S.D. (in vitro) or means ± S.E. (in vivo). Statistical significance was determined using student’s *t*-test and one-way analysis of variance (ANOVA), followed by post-hoc Duncan test (SPSS software ver. 20, Chicago, IL, USA). Values of *p* < 0.05 were considered statistically significant.

## Supplementary Materials

The following are available online, Figure S1: Structure of ginsenosides.
