# Supplementary material for: Red Ginseng Improves Exercise Endurance by Promoting Mitochondrial Biogenesis and Myoblast Differentiation
Source: Molecules. 2020 Feb 16;25(4):865. doi: 10.3390/molecules25040865 (PMC7070955; doi:10.3390/molecules25040865)
Supplement: Supplementary file 1 [file molecules-25-00865-s001.pdf]

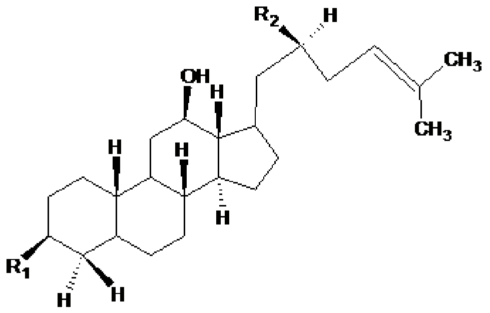

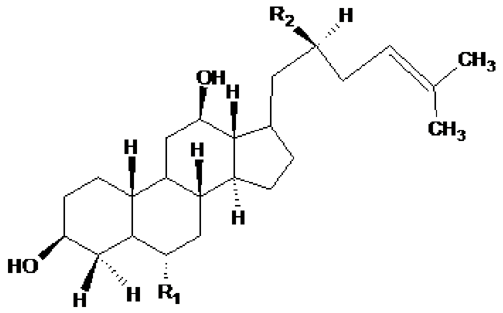

Ginsenosides	R1	R2
Rb1	Glc(2→1)Glc	Glc(6→1)Glc
Rb2	Glc(2→1)Glc	Glc(6→1)Ara(p)
Rb3	Glc(2→1)Glc	Glc(2→1)Xyl
Rc	Glc(2→1)Glc	Glc(6→1)Ara(f)
Rd	Glc(2→1)Glc	Glc
Rg3	Glc(2→1)Glc	H
Rh2	Glc	H

Ginsenosides	R1	R2
Re	Glc(2→1)Rha	Glc
Rg1	Glc	Glc
Rf	Glc(2→1)Glc	H
Rg2	Glc(2→1)Glc	H
Rh1	Glc	H

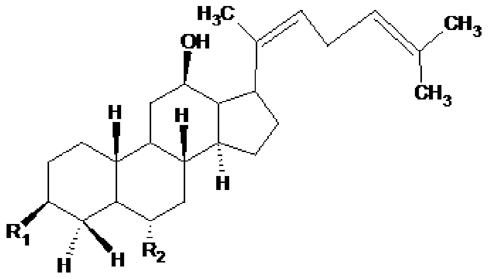

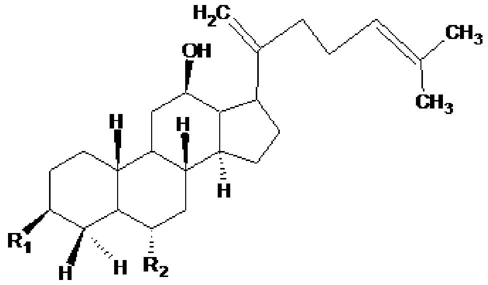

Ginsenosides	R1	R2
Rh4	H	Glc
Rg5	Glc(2→1)Glc	H

Ginsenosides	R1	R2
Rk1	Glc(2→1)Glc	H
Rk3	H	Glc

Supplementary Figure 1. Structure of ginsenosides.
